# Transgenic Production of an Anti HIV Antibody in the Barley Endosperm

**DOI:** 10.1371/journal.pone.0140476

**Published:** 2015-10-13

**Authors:** Goetz Hensel, Doreen M. Floss, Elsa Arcalis, Markus Sack, Stanislav Melnik, Friedrich Altmann, Twan Rutten, Jochen Kumlehn, Eva Stoger, Udo Conrad

**Affiliations:** 1 Leibniz Institute of Plant Genetics and Crop Plant Research (IPK), Plant Reproductive Biology, Corrensstr. 3, 06466, Stadt Seeland/OT Gatersleben, Germany; 2 Leibniz Institute of Plant Genetics and Crop Plant Research (IPK), Phytoantibodies, Corrensstr. 3, 06466, Stadt Seeland/OT Gatersleben, Germany; 3 Medical Faculty, Institute of Biochemistry and Molecular Biology II, Heinrich-Heine-University, Universitätsstrasse 1, 40225, Düsseldorf, Germany; 4 Department for Applied Genetics and Cell Biology, University of Natural Resources and Life Sciences, Muthgasse 18, 1190, Vienna, Austria; 5 Institute for Molecular Biotechnology, Biology VII, RWTH Aachen, Worringerweg 1, 52074, Aachen, Germany; 6 Department of Chemistry, University of Natural Resources and Life Sciences, Muthgasse 18, 1190, Vienna, Austria; 7 Leibniz Institute of Plant Genetics and Crop Plant Research (IPK), Structural Cell Biology, Corrensstr. 3, 06466, Stadt Seeland/OT Gatersleben, Germany; Wuhan University, CHINA

## Abstract

Barley is an attractive vehicle for producing recombinant protein, since it is a readily transformable diploid crop species in which doubled haploids can be routinely generated. High amounts of protein are naturally accumulated in the grain, but optimal endosperm-specific promoters have yet to be perfected. Here, the oat *GLOBULIN1* promoter was combined with the legumin B4 (LeB4) signal peptide and the endoplasmic reticulum (ER) retention signal (SE)KDEL. Transgenic barley grain accumulated up to 1.2 g/kg dry weight of recombinant protein (GFP), deposited in small roundish compartments assumed to be ER-derived protein bodies. The molecular farming potential of the system was tested by generating doubled haploid transgenic lines engineered to synthesize the anti-HIV-1 monoclonal antibody 2G12 with up to 160 μg recombinant protein per g grain. The recombinant protein was deposited at the periphery of protein bodies in the form of a mixture of various N-glycans (notably those lacking terminal N-acetylglucosamine residues), consistent with their vacuolar localization. Inspection of protein-A purified antibodies using surface plasmon resonance spectroscopy showed that their equilibrium and kinetic rate constants were comparable to those associated with recombinant 2G12 synthesized in Chinese hamster ovary cells.

## Introduction

The concept of molecular farming was first aired over 20 years ago, but despite its much trumpeted potential (see review by [1]), the first plant-derived products have only recently reached the market. Examples include recombinant human glucocerebrosidase synthesized in carrot suspension cells [2], and human growth hormone and cytokines synthesized in the barley grain [3]. Genes encoding various monoclonal antibodies have been successfully expressed in planta [4–7], and have been subjected to clinical trials [8]. These achievements clearly demonstrate that plant-based production systems have been recognized as alternative platform to microbial and mammalian cells with specific advantages including the lack of human pathogens [9]. Although transient transgene expression systems have attracted greater investment than stable ones, largely because the development effort is less demanding, currently marketed products have all been based on stably transformed plants or plant cells.

The cereal grain is a particularly attractive vehicle for producing recombinant protein, because its evolution as a protein storage organ assures an environment in which proteins remain stable for long periods without any need for refrigeration or aseptic conditions. The specific advantages of barley are its tolerance of a wide range of environmental conditions, its largely autogamous mode of reproduction, its G.R.A.S. (generally recognized as safe) status, its well established agronomy, and its ease of genetic transformation. Many conventional barley breeding programs use doubled haploid technology to drive homozygosity in elite germplasm [10], and the combination of this methodology with genetic transformation provides a highly convenient means to fix transgenes, as shown by [11] in tobacco.

The major barley grain storage proteins are the hordeins, which are transported first into the lumen of the endoplasmic reticulum (ER), and are ultimately deposited in the protein storage vacuole (PSV) [12, 13]. In barley 50% of the grain's protein are hordeins, while 75% of oat grain proteins are globulins[14]. The genes encoding such storage proteins have therefore become the prime targets for characterization of endosperm-specific promoters. Vickers *et al*. [15], in a comparison of the effectiveness of a number of endosperm-specific promoters to transiently express transgenes in both barley and wheat, were able to identify a cis-acting element, which made a substantial contribution to the level of transgene expression in the endosperm. Here the synthesis of an anti-HIV monoclonal antibody in the barley grain is described. The approach involved the use of the oat *GLO1* (*GLOBULIN1*) promoter to drive the transgene, and the addition of signal peptides to direct and stabilize the recombinant protein.

## Materials and Methods

### Plasmids, strains and generation of transgenic barley plants

The oat *GLO1* promoter sequence was amplified from the genomic DNA of oat cv. ‘Solidor’ (IPK Genebank accession AVE 1500) using the primer pair GH-AsGlo1-EcoRV-F1 and GH-AsGlo1-PstI-R1 (sequences given in S3 Table). The resulting 977 bp *Eco*RV/*Pst*I fragment was cloned into pCK21/4 (C. Kaydamov, unpublished) in front of the Legumin type B 5’UTR and signal peptide from *Vicia faba* fused to GFP and the endoplasmic reticulum retention signal KDEL (*LeB4* 5’UTR::*LeB4*SP:*gfp*:*KDEL*) cassette to produce the construct pGH220. An *Sfi*I fragment harboring the entire expression cassette was then subcloned into p6d35S (DNA Cloning Service, Hamburg, Germany) to generate the construct pGH223 (*d35S*P::*HPT-AsGLO1*P::*LeB4*SP:*gfp*:*KDEL*). Plasmids harboring the coding sequences for the 2G12 heavy chain (HC) and light chain (LC) were based on pGH221 which is the same as pGH220 but with bacterial chloramphenicol resistance. The primer pair LC-Nco/LC-Sal (S3 Table) was used to amplify the 2G12 LC sequence from a template of pRTRA 35S 2G12LC [10] and the resulting *Nco*I/*Sal*I fragment was substituted for *GFP* in pGH221 to form the construct pGH221-LC. Similarly, 2G12 HC was amplified using the primer pair HC-Nco/HC-Sal (S3 Table) from a template of pRTRA 35S 2G12HC [11], and the resulting *Nco*I/*Sal*I fragment introduced into pGH221 to generate pGH221-HC. Finally, the two *Sfi*I fragments were cloned into p6d35S to generate the binary vectors pGH248 (HC) and pGH249 (LC). A schematic representation of pGH223, pGH248 and pGH249 is given in S1 Fig. The binary plasmids were all introduced into *Agrobacterium tumefaciens* strain AGL1 [16], which was co-cultured with immature embryos of barley cv. ‘Golden Promise’, as described elsewhere [17].

### Embryo rescue and segregation for hygromycin resistance

Aseptically dissected immature embryos from 21–28 day old T_1_ or T_2_ caryopses were cultured for 24 h on B5 medium [18] to induce their germination. The germinating embryos were transferred onto K4N medium [19] supplemented with 100 mg/L hygromycin, and held for up to ten days at 24°C under a 16 h photoperiod.

### Generation of doubled haploid lines

Microspores at the highly vacuolated, pre-mitotic stage were isolated from immature spikes following [20]. The ploidy level of regenerants was determined using a Ploidy Analyser 1 (PARTEC, Muenster, Germany), following the manufacturer’s protocol. To induce whole genome duplication where it was not spontaneous, haploid plantlets were exposed to 0.1% w/v colchicine, following the [21] procedure.

### Grain protein extraction and the quantification of GFP

Soluble proteins were extracted from mature grains in 2% (w/v) SDS, 5% (v/v) β-mercaptoethanol, 0.001% (w/v) pyronin, 10% (v/v) glycerol, 0.063 M Tris-HCl [22]. Following their separation by SDS-PAGE, the proteins were transferred onto a membrane, which was challenged with a monoclonal anti-GFP mouse IgG_1_ antibody (Roche Diagnostics, Mannheim, Germany) and subsequently by an anti-mouse IgG (H+L) alkaline phosphatase conjugate antibody (Promega, Madison, WI, USA). Conjugated proteins were visualized by supplying Western Blue® Stabilized Substrate for Alkaline Phosphatase (Promega, Mannheim, Germany). GFP was quantified densitometrically using TotalLab™ software (TL120, Nonlinear Dynamics Ltd., Newcastle upon Tyne, UK).

For calculating the antibody yield in %TSP, 0.1 g grain flour was extracted with 400 μl extraction buffer and total protein content of the sample was estimated using standard methods.

### PCR and DNA gel blot analysis

The template used in PCRs amplifying from plant genomic DNA was extracted from ~100 mg snap-frozen leaf, following [23]. Each PCR was seeded with 100 ng template, using primers detailed in S3 Table. Amplicons were separated by agarose gel electrophoresis and visualized by ethidium bromide staining. DNA gel blot analysis were carried out following the [24] procedure, and were based on 30 μg aliquots of electrophoresed *Hind*III-digested genomic DNA transferred onto a positively charged nylon membrane (Roche Diagnostics, Mannheim, Germany). The membranes were hybridized with a DIG-dUTP-labeled *HPT* probe (Roche Diagnostics, Mannheim, Germany).

### Light and electron microscopy

Transverse median sections of mature barley grains were cut with a razor blade, and placed in a droplet of 50 mM phosphate buffer (pH 7.0) under a cover slip. Fluorescence microscopy was carried out using a LSM510 META confocal laser scanning microscope (Carl Zeiss, Jena, Germany). GFP fluorescence was analyzed with a 488 nm laser line in combination with a 505–530 nm band pass filter. Genuity of the GFP signal was confirmed by lambda stack analysis. Cell wall autofluorescence was captured with a 364 nm laser line in combination with a 385 nm long pass filter. For the localization of recombinant 2G12, thin slices were cut from transgenic and wild type endosperm using a razor blade, floated in 0.1 M phosphate buffer (pH 7.4), fixed in 4% (w/v) paraformaldehyde, 0.5% (v/v) glutaraldehyde in 0.1 M phosphate buffer (pH 7.4) and processed as described by [25]. Both 1 μm thick sections mounted on glass slides for fluorescence microscopy and sections showing silver interference colors mounted on gold grids for electron microscopy were pre-incubated in 5% (w/v) fraction V bovine serum albumin in 0.1 M phosphate buffer (pH 7.4) and then challenged with an recommended dilution of either polyclonal goat anti-HC or anti-LC (Sigma, Carlsbad, CA, USA), polyclonal rabbit anti-wheat gliadin (Sigma, Carlsbad, CA, USA) or polyclonal anti-PDI. The sections were then exposed to a solution of secondary antibody in 0.1 M phosphate buffer (pH 7.4). For fluorescence microscopy experiments, the secondary antibody was either donkey anti-goat IgG Alexa Fluor® 488 (Life Technologies, Carlsbad, USA) or donkey anti-rabbit IgG Alexa Fluor® 526 (Life Technologies, Carlsbad, USA), while for the electron microscopy experiments, the antibody was either rabbit anti-goat IgG 10 nm gold (British BioCell International, Cardiff, UK) or goat anti-rabbit IgG 10 nm gold (British BioCell International).

### Immunoblotting

Hordeins were extracted from 50 mg of flour milled from cv. Golden Promise grain in 1 mL 55% (v/v) isopropanol, 2% (v/v) β-mercaptoethanol for 1 h at 65°C. After centrifugation, a 50 μL aliquot was combined with 750 μL acetone, and the mixture was re-centrifuged. The resulting pellet was dissolved in 60 μL LDS buffer and loaded onto a 10% (w/v) SDS polyacrylamide gel. Bands corresponding to the B and C hordeins were identified according to [13]. Polyclonal rabbit anti-wheat gliadins serum (Sigma, Carlsbad, CA, USA) was used for detection. Total soluble proteins were extracted from individual grains in phosphate buffered saline (PBS; 1:3 w:v) and then centrifuged (17,500 x *g*, 4°C). For sequential extractions, the pellets were washed six times with PBS (NaCl 137 mM, KCl 2.7 mM, Na_2_HPO_4_ 10 mM, KH_2_PO_4_ 1.8 mM) as described above and the sixth supernatant was kept as a negative control. The pellet was then re-extracted in an equal volume 1x PBS supplemented with, 5% (v/v) β-mercaptoethanol, and 8 M urea. The resulting supernatant was boiled for 10 min, and then subjected to electrophoresis through a 12% (w/v) SDS-PAGE under reducing conditions. Immunoblotting analyses were carried out according to standard protocols.

### Biacore analysis

Binding ratios to bacterial surface proteins protein-A, protein-L and HIV glycoprotein with a molecular weight of 120 kDa (gp120) were determined on a BiacoreT200 instrument (GE Healthcare, Braunschweig, Germany) using HBS-EP as running buffer and assay conditions reported previously [5]. The antigen binding kinetics was measured using a protein-A capture assay [11, 26]. HIV-1 gp120 was obtained from the Center of AIDS Reagents (CFAR/NIBSC, Potters Bar, Hertfordshire, UK) and CHO-derived 2G12 was provided by Polymun Scientific GmbH (Klosterneuburg, Austria).

### Glycosylation

The N-glycan analysis was applied to tryptic glycopeptides, as described by [27]. Briefly, the heavy chain of the SDS-PAGE separated IgG was excised from an SDS-PAGE gel, S-alkylated, digested with sequencing grade modified trypsin (Promega, Mannheim, Germany) and subsequently separated through a reverse phase capillary column (Biobasic C18, ThermoScientific, Vienna, Austria) and a Q-TOF Ultima mass spectrometer (Waters, Manchester, UK).

## Results

### The oat *GLOBULIN1* promoter produces endosperm-specific gene expression

**Fig 1:**
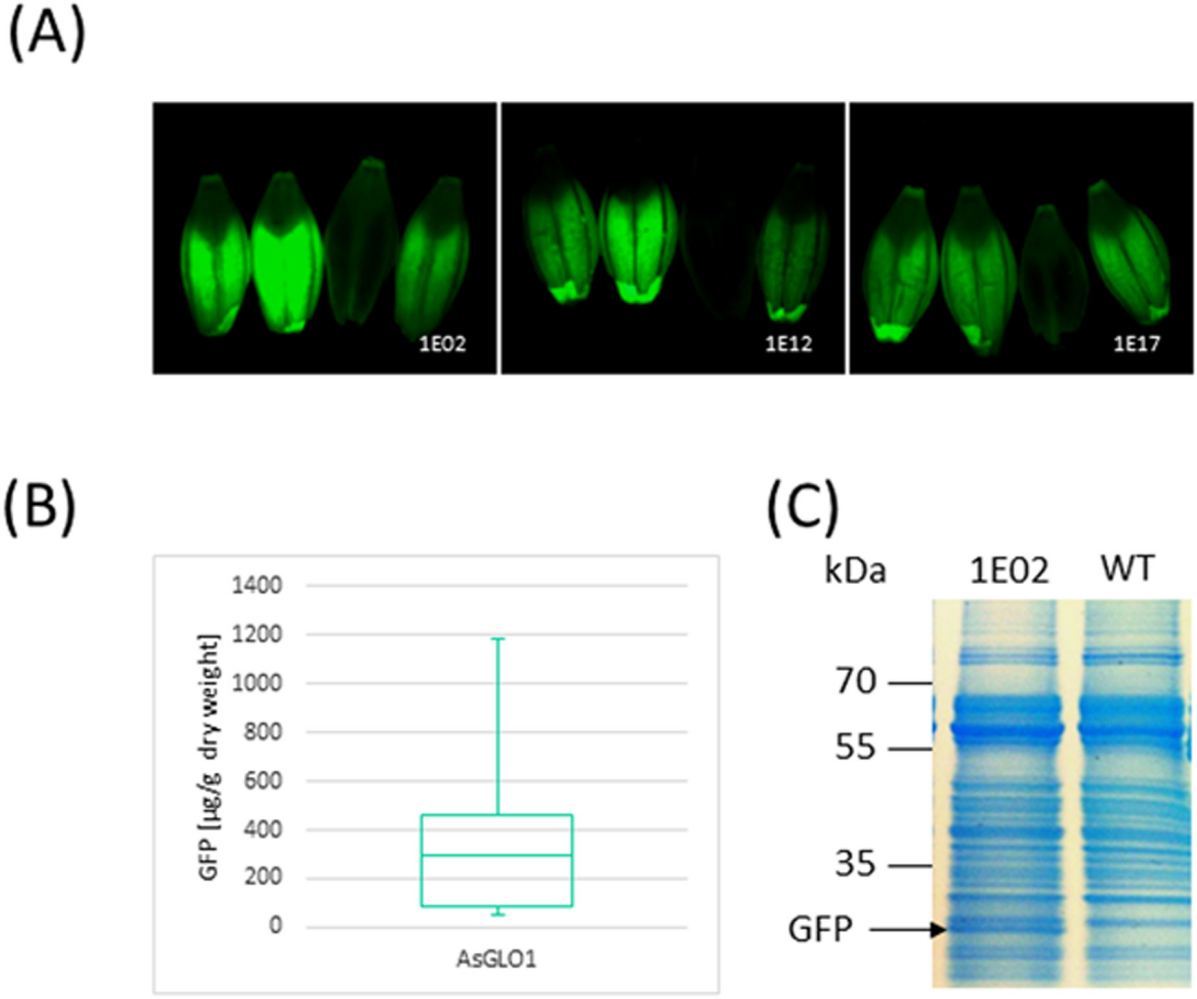
Establishment of an endosperm transgene expression system. (A) Fluorescence microscopy of four grains expressing *GFP* driven by the oat *GLO1* promoter. (B) Quantification of GFP based on Western blotting with an anti GFP antibody. (C) Total soluble protein profile obtained by Coomassie Brilliant Blue staining of 12% SDS-PAGE separations of extracts of a T_3_ selection of line BG136/1E02-14-4 expressing *GFP*.

A set of 50 independent primary transgenics harboring pGH223 (*d35S*P::*HPT-AsGLO1*P::*LeB4*SP:*gfp*:*KDEL*) was generated (S1 Fig). Inspection of T_1_ mature grains revealed a variable level of GFP accumulation in the endosperm, but no case of any detectable signal in the embryo (Fig 1A). In the subsequent generation, GFP was accumulated in the grain to a level of up to 1.2 mg per g dry weight (Fig 1B). GFP could also be detected by Coomassie Brilliant Blue staining of SDS-PAGE separations of total protein extracted from the grain (Fig 1C), a result which enabled a simple means to confirm the stable inheritance and expression of the transgene. The number of transgene copies introduced in each transgenic event was determined by DNA gel blot hybridization, which was also informative with respect to the zygosity (hemi- vs homo-) of the transgene(s) (S1 Table). The accumulation of GFP did not vary significantly between the single and multicopy transgene homozygotes, lying in the range 598–808 μg per g dry weight.

### The site of GFP deposition in the grain of transgenic plants

**Fig 2:**
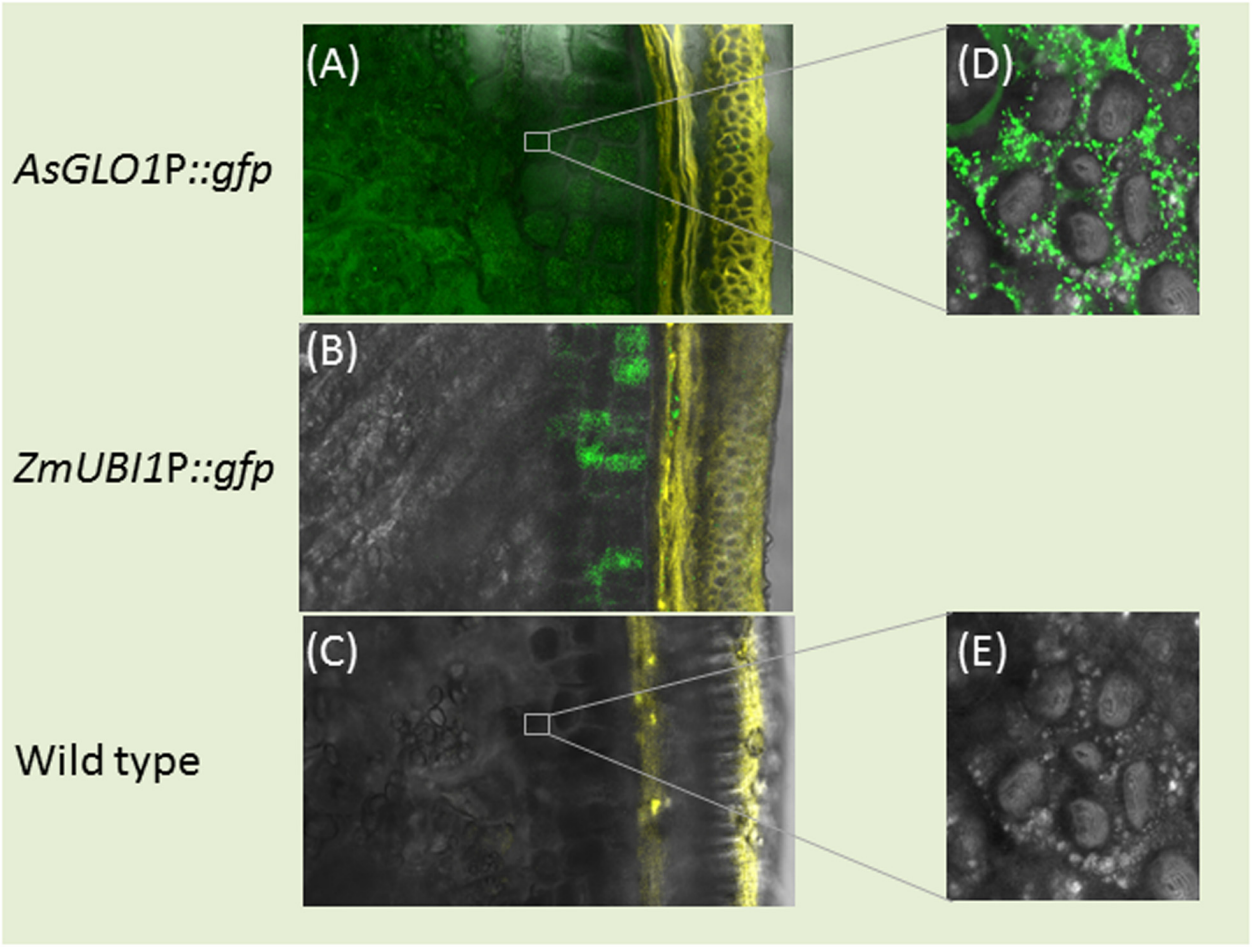
Transverse grain sections demonstrating the site of GFP accumulation (green) and cell wall autofluorescence (yellow). (A) GFP accumulation throughout the aleurone and endosperm of grains set by plants harboring the *AsGLO1*P::*GFP* transgene. (B) In a transgenic barley line constitutively expressing *GFP* (*ZmUBI1*P::*GFP*), GFP fluorescence was restricted to the aleurone layer. (C) In a wild type grain, no GFP signal was detectable. (D) Higher magnification of an endosperm cell reveals discrete deposits of GFP within the cytoplasm of the transgenic line shown in (a). (E) The cytoplasm of a wild type endosperm cell lacks any GFP signal. Bar: 50 μm in (A-C), 10 μm in (D-E).

Confocal laser scanning microscopy (CLSM) of mature grains set by the transgenic plant 1E17 (harboring the construct *d35S*P::*HPT-AsGLO1*P::*LeB4*SP:*gfp*:*KDEL*) confirmed an accumulation of GFP in both aleurone layer and starchy endosperm (Fig 2A). In the grains set by a homozygous selection of plant B13/10E28 (which expressed *GFP* under the control of the maize *UBIQUITIN1* promoter), GFP was abundant in the aleurone layer but hardly detectable in the starchy endosperm (Fig 2B and 2C). At the cellular level the GFP signal was uniformly distributed throughout the cytoplasm of endosperm cells, consistent with its deposition to small, probably vesicle-like compartments (Fig 2D). Wild type grains lacked any GFP activity (Fig 2E).

### Stable barley transgenic lines expressing 2G12 in the endosperm

**Fig 3:**
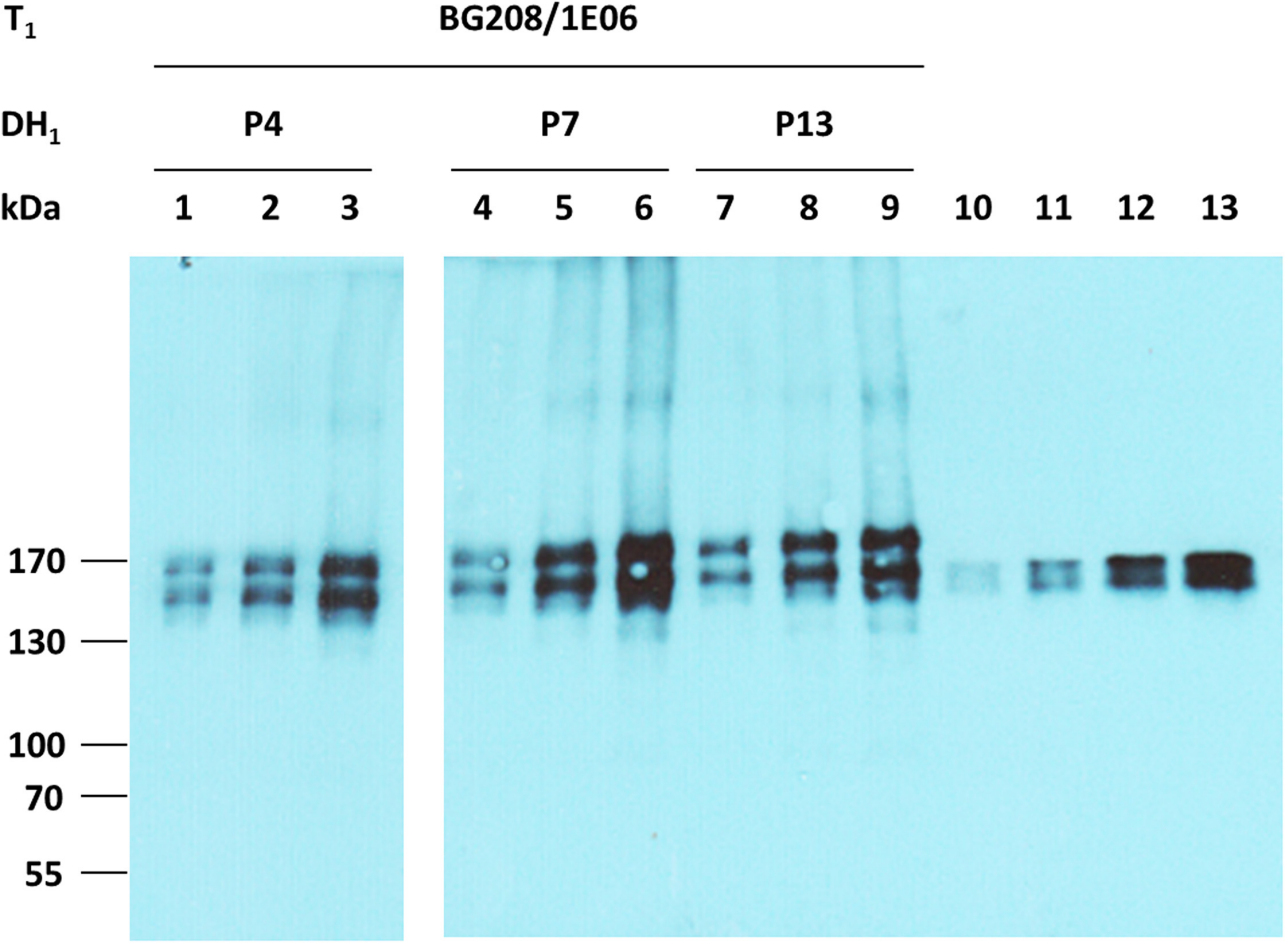
Detection of 2G12 antibodies in grain extracts. Grain extracts and CHO cell-derived 2G12 standards were separated by gradient SDS-PAGE under non-reducing conditions. 2G12 antibodies were detected by their binding to anti-human IgG Fcg-peroxidase. Lanes 1–3: 0.5, 1.0 and 2.0 μg of protein extracted from BG208/1E06-P4. Lanes 4–6: 0.5, 1.0 and 2.0 μg of protein extracted from BG208/1E06-P7. Lanes 7–9: 0.5, 1.0 and 2.0 μg of protein extracted from BG208/1E06-P13. Lanes 10–13: 0.5, 1.0, 2.0 and 4.0 ng 2G12 obtained from CHO cells.

**Fig 4:**
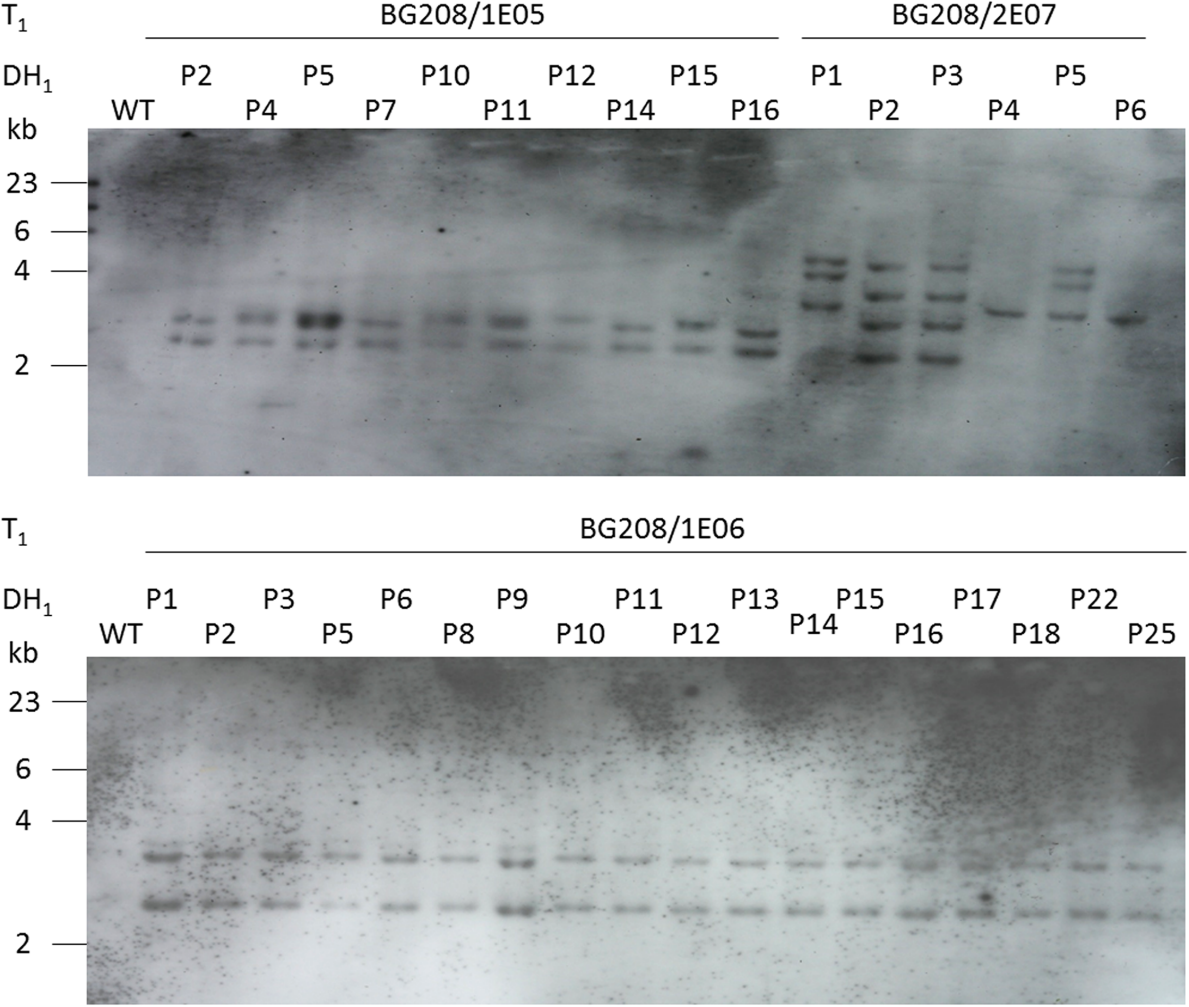
DNA gel blot analysis profiles of doubled haploid lines expressing the 2G12 monoclonal antibody. A 30 μg aliquot of genomic DNA was digested with *Hin*dIII, separated through a 0.8% agarose gel, blotted onto a Nylon membrane and hybridized with *HPT*. WT: wild type.

Transgenic barley plants harboring both the coding sequence for the heavy (HC) and light (LC) chains of the anti-HIV monoclonal antibody 2G12, driven by the oat *GLO1* promoter and including both the LeB4 signal peptide and SEKDEL retention signal (S1 Fig) were generated using the co-transformation strategy described by [28]. The inoculation of 180 immature embryos with an *Agrobacterium tumefaciens* strain harboring both pGH248 (LC) and pGH249 (HC) produced 29 regenerants. A multiplex PCR designed to reveal the presence of both transgenes identified six of these 29 plants as co-transformants, with ten carrying only the HC gene and 13 only the LC gene. A Western blot analysis suggested some variation in the amount of recombinant protein accumulated (data not shown). Transgene homozygotes were generated from three of the primary HC/LC carriers (BG208/1E05, BG208/1E06 and BG208/2E07) via pollen embryogenesis. Confirmed spontaneously doubled haploid regenerants carrying both transgenes were obtained from both BG208/1E05 (six lines) and BG208/1E06 (11 lines); the six regenerants from BG208/2E07 did not spontaneously undergo whole genome duplication, so required a colchicine treatment to restore self fertility. A sample of progeny of each doubled haploid line was tested for the expression of the two 2G12 antibody genes and the quantity of recombinant protein present in the grain was assessed. No free light or heavy chains were detected in Western blots (Fig 3 and data not shown). The proportion of total soluble grain protein represented by 2G12 lay between 0.1 to 0.4% (S2 Table). A DNA gel blot hybridization-based check of the transgene copy number in each line showed that both BG208/1E05 and BG208/1E06 harbored a single copy of both the HC and the LC genes (Fig 4). The transgenes strictly co-segregated, indicating a single integration event. The number of hybridizing fragments revealed in the BG208/2E07 material suggested the integration of six distinct transgenes in the T_0_ plant.

### Recombinant 2G12 antibody was deposited in endosperm protein bodies

**Fig 5:**
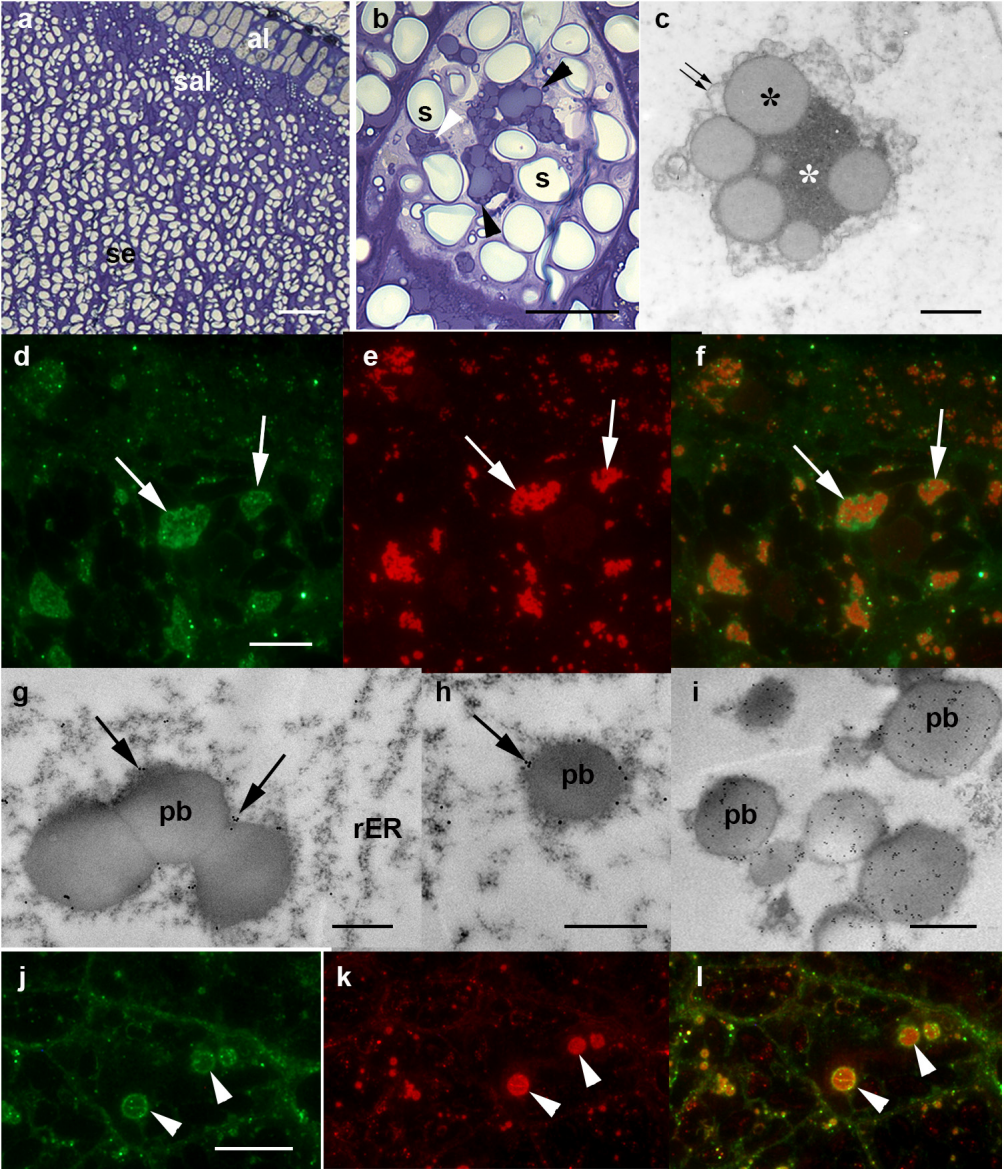
Deposition of 2G12 antibody in the barley endosperm. (A-C) Wild type grain, (D-L) transgenic grains. (A) Light microscopy shows three distinct layers, namely the aleurone layer (al), the sub-aleurone layer (sal) and the endosperm (se). (B) Toluidine blue staining of a mid-development sal cell. Note the oblong starch grains (s) and the large protein bodies (black arrowheads). The protein bodies are multiphasic and take up the stain differentially (white arrowheads). (C) Transmission electron microscopy, general, non-specific contrasting. The protein bodies lie within a membrane (double arrow) displaying their multiphasic nature: moderately electron-dense, spherical protein bodies (black asterisks) embedded in a highly electron-dense, amorphous matrix (white asterisk). (D-F) Fluorescence microscopy demonstrates the co-localization of 2G12 (D, arrows) and hordeins (E, arrows). The antibody chains accumulate in the periphery of the protein bodies alongside the hordeins (F, merged, arrows). (G-I) immuno-electron microscopy-based localization of 2G12. Label (arrowed) associated with the periphery of the protein bodies (pb). (I) Localization of PDI within the protein bodies (pb) that appear evenly labelled. (J-L) Co-localization of 2G12 antibody (J, arrowheads) and PDI (K, arrowheads). 2G12 was detected exclusively in the periphery of the protein bodies, labelled with PDI (L, merged, arrowheads). Bars: 20 μm in (A), 5 μm in (B, D-F, J-L), 1 μm in (C, G-I).

The pattern of endosperm maturation allows for cells at a variety of developmental stages to be visualized within a given section. Cells in the sub-aleurone layer (sal) form later than those in the inner part of the endosperm and have a higher relative protein content (Fig 5A and 5B). At the mid-developmental stage, the barley protein bodies harbored low electron density spherical protein inclusions embedded within an electron dense protein matrix (Fig 5C). The former contains B, C, γ1 and γ2 hordeins [29], and can be stained with toluidine blue (Fig 5B). The subcellular distribution of the recombinant protein in the sal of BG208/1E05 grain was compared with that of the hordeins; the latter were identified by an antibody which recognizes B and C hordeins [30] and thereby identifies protein bodies in the barley endosperm (S2 Fig). The analysis revealed that, although associated with the protein bodies, the recombinant 2G12 did not co-localize with the hordeins, but rather accumulated at the periphery of the hordein aggregates (Fig 5D–5F). This pattern of deposition was confirmed by immunoelectron microscopy, which detected label exclusively along the periphery of the developing protein bodies (Fig 5G and 5H). Given the recombinant 2G12 protein was engineered to carry a KDEL tag to encourage its retention in the ER, a comparison was made with the subcellular localization of protein disulphide isomerase (PDI), an endogenous HDEL-containing, ER-resident endosperm protein [31]. PDI was detectable in the ER and was evenly distributed within the protein bodies (Fig 5I and 5K), and there was no discernible effect on this distribution induced by the presence of the 2G12 transgenes (data not shown). The 2G12 and PDI deposition sites were non-identical: they occupied two distinct phases inside and along the margin of the protein bodies (Fig 5J and 5L).

### N-glycosylation of the recombinant 2G12 antibody

**Fig 6:**
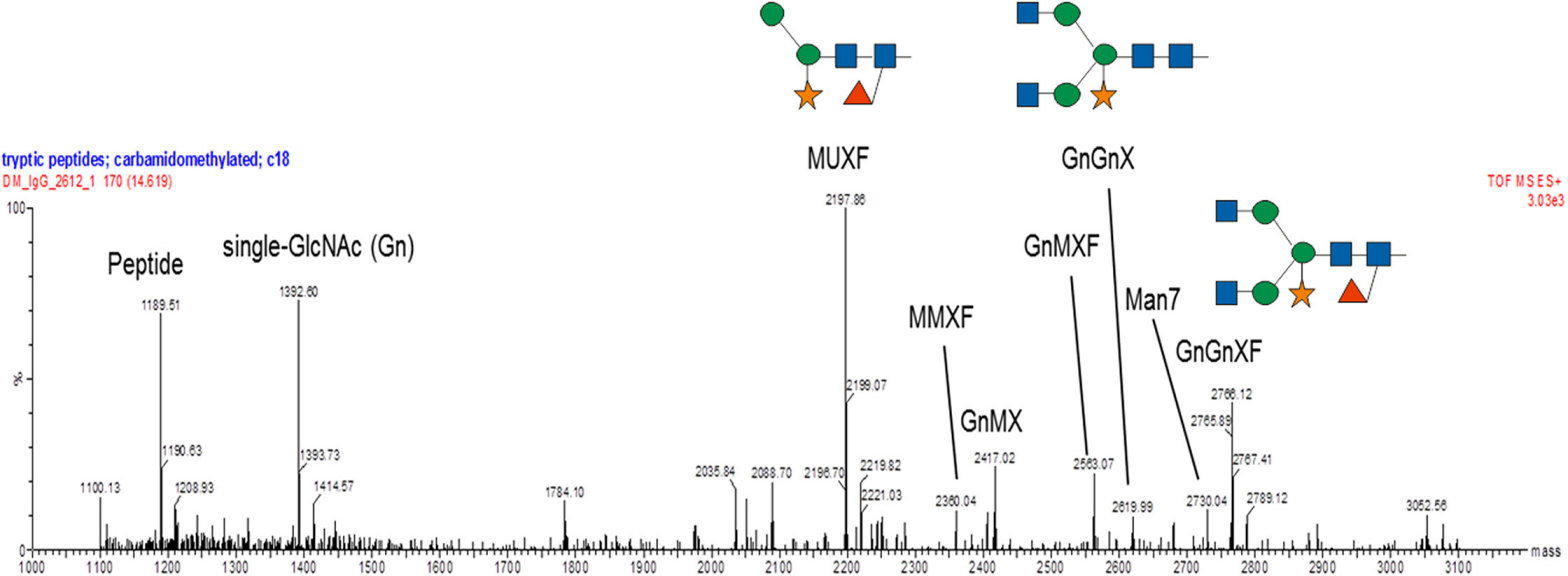
N-glycan analysis of grain extracts. The deconvoluted spectrum of the elution region of tryptic peptides and glycopeptides of the Fc peptide EEQYNSTYR shows the presence of a non-glycosylated peptide, carrying only a single GlcNAc residue. Peak heights roughly reflect the relative molar ratios. So smaller species may be somewhat over-represented. Abbreviations follow the proglycan system. GlcNAc, Man, Xyl and Fuc are represented, respectively, by blue squares, green circles, orange stars and red triangles.

An analysis of BG208/1E05 grains revealed the presence of a mixture of N-glycan structures (Fig 6). The main peak corresponded to a trimmed glycan lacking terminal N-acetyl glucosamine residues and carrying a β(1,2)-linked xylose core with an α(1,3)-linked fucose residue. This structure is commonplace in vacuolar glycoproteins and is indicative of the passage of the recombinant protein through the Golgi apparatus *en route* to the storage vacuole. A smaller quantity of complex N-glycans possessing terminal N-acetylglucosamines was also detected, diagnostic of glycoproteins which have been exported from the Golgi apparatus or secreted into the apoplast. The presence of significant amounts of this N-glycan structure is quite unusual in starchy cereal endosperm. The proportion of mannosidic N-glycans was very low, whereas structures consisting of just a single GlcNAc residue were abundant. This pattern is consistent with the trimming of oligomannosidic glycans by endoglycosidase activity, as has been observed in both the maize [5, 32] and the rice [33] endosperm. A significant quantity of non-glycosylated HC protein was also detected.

### The assembled recombinant 2G12 antibody is functional in a Biacore antigen binding assay

A comparison between barley and Chinese hamster ovary (CHO) recombinant 2G12 was based on a binding ratio assay under mass-transport limiting conditions [5]. Various dilutions of the antibody were passed over high-capacity protein-A (Fc2), protein-L (Fc3) and gp120 (Fc4) surfaces, the blank subtracted binding signals were plotted against one another and the binding ratios were then determined by linear regression. A relative binding ratio was derived by dividing the barley 2G12 value by the CHO 2G12 one. Whereas the relative protein-L-to-protein-A binding ratio was 100.6±0.8% (i.e., the proteins were essentially identical), the relative HIV-1 ^BaL^gp120-to-protein-A and HIV-1 ^BaL^gp120-to-protein-L binding ratios were, respectively, 87.5±1.1% and 87.0±0.6%, indicating that the barley recombinant antibody included an inactive fraction which did not bind the antigen as effectively. The binding kinetics were explored in more detail using a protein-A capture assay based on monomeric soluble HIV-1 ^BaL^gp120 (EVA607, CFAR/NIBSC, Potters Bar, Hertfordshire, UK) as the analyte. The analysis compared two different batches of both CHO- and barley-derived 2G12, along with aliquots sampled from a single barley 2G12 preparation stored at either -20°C or +4°C. The samples were diluted to ensure that they bound immobilized protein-A equivalently (166–174 RU), with the remaining minor differences corrected by normalizing the binding curves to 170 RU. The resulting binding curves were fitted using a simple 1:1 binding model (Table 1). Only minor differences were observed in the kinetic rate constants (*k*_ass_ and *k*_diss_), the equilibrium dissociation constant *K*_D_ and the fitted *R*_max_ values, thus the binding behaviour of the antibodies from plants and CHO cells is principally comparable.

**Table 1:** Summary of kinetic analysis.

Sample	*k*_ass_ [1/Ms]	k_diss_ [1/s]	*K*_D_ [nM]	*R*_max_ [RU]	Chi^2^	U
^barley^2G12 (4°C)	8.57∙10^4^	1.33∙10^−4^	1.55	63.1	1.68	7
^barley^2G12 (-20°C)	9.15∙10^4^	1.28∙10^−4^	1.40	65.4	1.50	7
^CHO^2G12 i-8 (older batch)	10.4∙10^4^	1.19∙10^−4^	1.14	65.5	2.08	9
^CHO^2G12 A1 (newer batch)	10.2∙10^4^	1.44∙10^−4^	1.41	61.9	1.87	7

## Discussion

To date, a number of endosperm-specific expression systems have been developed, exploiting the promoters of genes encoding a variety of endosperm storage proteins from barley, wheat and rice (for review see [34]). Few attempts have been made to compare their effectiveness based on the quantification of reporter protein abundance. Patel *et al*. [35] showed that the transgenic expression in barley of the bacterial gene *XynA* was fivefold greater when driven by the *GluB-1* (from rice) promoter than it was by the *Hor2-4* (from barley) promoter, and were able to recover a maximum yield of recombinant protein equivalent to 0.037% of the total grain protein. Meanwhile, Vickers *et al*. [15] expressed *GFP* in barley driven by the oat *GLO1* promoter, and achieved a yield equivalent to 10% of the total grain protein. The *GLO1* promoter was also employed in the present study, with the inclusion of signal sequences derived from LeB4 to encourage uptake of the transgene product into the ER and KDEL for its retention there. The 2G12 HC and LC coding sequences used here have previously been introduced as transgenes into tobacco, maize and rice [4–6, 33], although not with the same promoters as used here. The proportion of the recombinant antibody found in protein extracts of the tobacco leaf and seed varied from 0.3–0.4%, raised to about 1% by incorporating elastin-like peptide (ELP) fusion technology [11]. In maize, the rice *GLUTELIN1* promoter achieved the accumulation of up to 75 μg/g 2G12 in the grain [6]. The range of 2G12 accumulation observed here in the barley grain was 0.1–0.4% of TSP corresponding to 33–160 μg/g grains (Table 2), in line with the amounts of an anti-glycophorin single chain antibody fused to an HIV epitope and recovered from barley grains by [36]. To compare the barley grain production system with other production platforms based on stable nuclear transgenic plants, we have reproduced published data referring to antibody 2G12 in Table 2. For stable tobacco-based systems, we refer to the report of Sack et al. [37]. Higher yields have been achieved with transient expression systems based on Agro-infiltration of N. benthamiana leaves [25, 38]. However, the specific amount of the transgenic protein produced per biomass unit is only one parameter that can be used to describe the productivity of an expression system. More meaningful assessments will also include the space, time and labor input required for completing a production cycle, as well as the flexibility in terms of upscaling and batch processing. Greenhouse-based production of 2G12 in barley grains will result in 1 g antibody per 10 square meters. With three possible harvests per year a 120 square meter greenhouse would be sufficient to produce 36 g of antibody 2G12. This is similar to the figures calculated for stable transgenic tobacco [36]. A main benefit of the barley grain production system is the stability of antibodies in seeds allowing conventional harvesting and storage at ambient conditions. The potential of an in planta recombinant antibody production system using cereal crops has yet to be fully exploited. Considerations also have to comprise regulatory issues including the definition of master seed banks, maintenance of pure genotypes and the quality control procedures for the recombinant antibody [38, 39]. Biosafety issues are closely linked to the level of containment and have been discussed elsewhere [40].

**Table 2:** Comparison of different stable transgenic expression systems for the production of 2G12 mAB in plants.

Species	Tag	Organ	Promoter	Yield		Reference
Tobacco	-	Leaf	CaMV *35S*	17–18 μg/mL	(45 μg/g)*	[37]
	KDEL KDELELP, KDEL	leafseedLeaf	CaMV *35S* Faba bean *USP*CaMV *35S*	0.4% of TSP0,3% of TSP1% of TSP	(2.8 μg/g)**(1.7 μg/g)**(6.9 μg/g)**	[11][11][11]
	ELP, KDEL	Seed	Faba bean *USP*	1% of TSP	(5.8 μg/g)**	[11]
Maize	KDEL-	Kernels/ endospermKernels/ endosperm	Rice *Glut1*Rice *Glut1*	30 μg/g75 μg/g		[5][6]
Rice	-	Kernels/ endosperm	Rice *Glut1*	46 μg/g		[33]
Barley	KDEL	Grain/ endosperm	Oat *Glo1*	0.4% of TSP	(160 μg/g)	this study

* line selected over several generations and optimized conditions for growth and harvest

**units were converted based on the average TSP values obtained with the respective extraction method [10]

Barley doubled haploids can be produced either via the pollen embryogenesis or the gynogenesis (following wide crosses with *H*. *bulbosum*) pathway [10]. The major feature of doubled haploidy is that genotypes become fixed in just a single recombination step. In the doubled haploid lines BG208/1E05 and BG208/1E06, the two HC and LC 2G12 genes were co-inherited, as shown by an identical integration footprint for the two genes. This was not the case for the BG208/2E07 material, where the number of transgene copies present varied, indicating a lack of linkage. As might be expected from highly homozygous material, there was little plant to plant variation in the yield of 2G12 antibody, fulfilling the need to breed material which delivers a stable and predictable level of transgene product as prerequisite for molecular farming. 2G12 is planned to be administered not by injection but by vaginal application to prevent reinfections. This fact minimizes the risk for allergic reactions caused by crop-based allergens. Future scale up and downstream processing development will be required to meet the standards for therapeutic proteins and should include the removal of hordeins by (“polishing” with) size exclusion chromatography and ion exchange chromatography. Despite the inclusion of a KDEL tag on both 2G12 antibody chains, the recombinant protein accumulated alongside the hordeins within the PSV. The deposition of KDEL-tagged proteins in this site has been noted previously by [25]. Cereal prolamins are thought to be routed directly from the ER to the vacuole, bypassing the Golgi apparatus [41]. Thus it seems possible that recombinant proteins engineered to accumulate in the ER may be passively drawn into the PSV by the bulk flow of prolamins. However, the deposition of 2G12 was largely within the periphery of the protein bodies which harbor the hordeins, unlike that of the KDEL-tagged recombinant human serum albumin expressed in wheat, which was rather evenly dispersed within the prolamins [25]. The major 2G12 deposition site coincided with that of hordein γ1 and γ2, both of which have the capacity to form intermolecular disulphide bonds [29]. A portion of the antibody chains of recombinant 2G12 generated in the maize kernel remained insoluble, ostensibly due to their covalent interaction with zein [42]. It may therefore be that the antibody chains can form hetero-oligomers with hordein γ1 and γ2, since this would explain the observed localization of 2G12. However, unlike in the maize situation, there was no evidence of any insoluble/covalently linked antibody (data not shown). The localization of 2G12-KDEL within the barley PSV also contrasts with its predominant localization within the maize ER-derived zein bodies [5]. Note, however, that PSVs are less abundant in the maize endosperm than in the endosperm of other cereals, especially at later developmental stages [32].

The N-glycan profile of 2G12-KDEL included a major proportion of structures consisting of just a single GlcNAc residue. The phenomenon likely reflects the presence of endo-N-acetyl-beta-D-glucosaminidase activity in the barley grain [43]. Maize grain and tobacco seed-derived KDEL-tagged 2G12 antibody harbored mainly oligomannosidic N-glycans and single GlcNAc residues, and only a small proportion of complex glycan structures [5, 11]. The most abundant N-glycan structure in the barley 2G12 product was a paucimannosidic glycan with Golgi apparatus-specific modifications (MUXF), typical of vacuolar localized proteins. While still consistent with its deposition in the PSV, this glycan pattern is somewhat unusual for a KDEL-tagged protein, as it suggests that a major part of it must have passed through the Golgi apparatus *en route* to the PSV. Nevertheless, the KDEL sequence was not removed from the protein (data not shown). The transgene construct N-terminal signal peptide sequence used for barley transformation replaced the native IgG sequence used for maize with the LeB4 signal sequence. Furthermore, the KDEL sequence was attached in a different manner, since an extra four residues were added upstream of the SEKDEL sequence in the barley transgene. It is possible that one or both of these differences may have interfered with trafficking or with the exposure of the KDEL signal.

A higher proportion of the 2G12-KDEL product remained non-glycosylated in the barley product than in the maize one (11%, see [6]). The equivalent proportion in rice was even higher [33], as it was also in *Arabidopsis thaliana* seeds producing high amounts of a scFvFc antibody; the potential explanation in the latter case was an overloading of the glycosylation machinery [44]. The equilibrium and kinetic rate constants of the barley 2G12-KDEL and the CHO 2G12 antibodies were highly comparable, with the differences lying well within the range observed between different antibody production batches. This is in agreement with earlier analyses of 2G12 which have suggested that its functionality is independent of both the glycoform and source of production [5, 6, 11, 33, 37].

In summary, the endosperm-specific expression system based on the oat *GLO1* promoter in combination with the LeB4 signal peptide has been shown to be effective in barley, and is appropriate as a technology for producing recombinant protein in the barley grain. The test protein, an anti-HIV antibody, was produced in a functional and soluble form and accumulated within the periphery of the PSV, alongside the hordeins. Its level of accumulation exceeded those achieved in transgenic tobacco, maize and rice.

## Supporting Information

S1 Fig Schematic representation of the T-DNAs involved in the binary vectors used for barley transformation. (DOCX)

S2 Fig Sites of hordein deposition in the barley endosperm. (TIF)

S1 Table Transgene segregation in three T_2_ barley populations, based on the expression of *HPT* in germinating immature embryos and the quantification of GFP in the grain. (DOCX)

S2 Table Quantification of 2G12 antibody in the doubled haploid lines. (DOCX)

S3 Table Primer sequences used for the identification of T-DNA elements. (DOCX)

## References

[1] Fischer R, Buyel JF, Schillberg S, Twyman RM. Molecular Farming in Plants: The Long Road to the Market In: Howard JA and Hood EE, editors. Commercial Plant-Produced Recombinant Protein Products. Biotechnology in Agriculture and Forestry 68 Berlin Heidelberg: Springer; 2014 pp. 27–41.

[2] Aviezer D, Brill-Almon E, Shaaltiel Y, Hashmueli S, Bartfeld D, Mizrachi S, et al A plant-derived recombinant human glucocerebrosidase enzyme—a preclinical and phase I investigation. PloS ONE 2009; 4: e4792 10.1371/journal.pone.0004792 19277123 PMC2652073

[3] Magnusdottir A, Vidarsson H, Björnsson JM, Örvar BL. Barley grains for the production of endotoxin-free growth factors. Trends in Biotechnol. 2013; 31: 572–580. 10.1016/j.tibtech.2013.06.002 23849675

[4] Floss DM, Sack M, Stadlmann J, Rademacher T, Scheller J, Stöger E, et al Biochemical and functional characterization of anti-HIV antibody-ELP fusion proteins from transgenic plants. Plant Biotechnol J. 2008; 6: 379–391. 10.1111/j.1467-7652.2008.00326.x 18312505

[5] Rademacher T, Sack M, Arcalis E, Stadlmann J, Balzer S, Altmann F, et al Recombinant antibody 2G12 produced in maize endosperm efficiently neutralizes HIV-1 and contains predominantly single-GlcNAc N-glycans. Plant Biotechnol J. 2008; 6: 189–201. 17979949 10.1111/j.1467-7652.2007.00306.x

[6] Ramessar K, Capell T, Christou P. Molecular pharming in cereal crops. Phytochem Rev. 2008; 7: 579–592.

[7] Strasser R, Stadlmann J, Schähs M, Stiegler G, Quendler H, Mach L, et al Generation of glyco-engineered *Nicotiana benthamiana* for the production of monoclonal antibodies with a homogeneous human-like N-glycan structure. Plant Biotechnol J. 2008; 6: 392–402. 10.1111/j.1467-7652.2008.00330.x 18346095

[8] Ma J, Drossard J, Lewis D, Altmann F, Boyle J, Christou P, et al Regulatory approval and a first-in-human phase I clinical trial of a monoclonal antibody produced in transgenic tobacco plants. Plant Biotechnol J. 2015 10.1111/pbi.12416 26147010

[9] De Muynck B, Navarre C, Boutry M. Production of antibodies in plants: status after twenty years. Plant Biotechnol. J. 2010; 8: 529–563. 10.1111/j.1467-7652.2009.00494.x 20132515

[10] Kumlehn J. Haploid technology In: Kumlehn J, Stein N, editors. Biotechnological approaches to barley improvement. Biotechnology in agriculture and forestry 69 Berlin: Springer; 2014 pp. 379–392.

[11] Floss DM, Sack M, Arcalis E, Stadlmann J, Quendler H, Rademacher T, et al Influence of elastin-like peptide fusions on the quantity and quality of a tobacco-derived human immunodeficiency virus-neutralizing antibody. Plant Biotechnol J. 2009; 7: 899–913. 10.1111/j.1467-7652.2009.00452.x 19843249

[12] Cameron-Mills V. The structure and composition of protein bodies purified from barley endosperm by silica sol density gradients. Carlsberg Res Commun. 1980; 45: 557–576.

[13] Cameron-Mills V, von Wettstein D. Protein body formation in the developing barley endosperm. Carlsberg Res Commun. 1980; 45: 577–594.

[14] Shewry PR. Improving the protein content and composition of cereal grain. J Cereal Sci. 2007; 46: 239–250.

[15] Vickers CE, Xue G, Gresshoff PM. A novel cis-acting element, ESP, contributes to high level endosperm-specific expression in an oat globulin promoter. Plant Mol Biol. 2006; 62: 195–214. 16915522 10.1007/s11103-006-9014-1

[16] Lazo GR, Stein PA, Ludwig RA. A DNA transformation-competent Arabidopsis genomic library in *Agrobacterium* . Biotechnol. (N. Y.) 1991; 9: 963–967. 10.1038/nbt1091-963 1368724

[17] Hensel G, Valkov V, Middlefell-Williams J, Kumlehn J. Efficient generation of transgenic barley: the way forward to modulate plant-microbe interactions. J Plant Physiol. 2008; 165: 71–82. 17905476 10.1016/j.jplph.2007.06.015

[18] Gamborg O, Miller R, Ojima K. Nutrient requirement of suspension cultures of soybean root cells. Exp Cell Res. 1968; 50: 151–158. 5650857 10.1016/0014-4827(68)90403-5

[19] Kumlehn J, Serazetdinova L, Hensel G, Becker D, Loerz H. Genetic transformation of barley (*Hordeum vulgare* L.) via infection of androgenetic pollen cultures with *Agrobacterium* tumefaciens. Plant Biotechnol J. 2006; 4: 251–261. 17177801 10.1111/j.1467-7652.2005.00178.x

[20] Coronado MJ, Hensel G, Broeders S, Otto I, Kumlehn J. Immature pollen-derived doubled haploid formation in barley cv. Golden Promise as a tool for transgene recombination. Acta Physiol Plant. 2005; 27: 591–599.

[21] Thiebaut J, Kasha KJ. Modification of the colchicine technique for chromosome doubling of barley haploids. Can J Genet Cytol. 1978; 20: 513–521.

[22] Popelka JC. Development of a genetic transformation protocol for rye (*Secale cereale* L.) and characterisation of transgene expression after biolistic or *Agrobacterium*-mediated gene transfer. Dissertation, University of Halle, 2002; http://sundoc.bibliothek.uni-halle.de/diss-online/02/02H059/index.htm

[23] Pallotta MA, Graham RD, Langridge P, Sparrow DHB, Barker SJ. RFLP mapping of manganese efficiency in barley. Theor Appl Genet. 2000; 101: 1100–1108.

[24] Sambrook J, Fritsch EF, Maniatis T. Molecular cloning—a laboratory manual New York: Cold Spring Laboratory Press, 1989.

[25] Arcalis E, Marcel S, Altmann F, Kolarich D, Drakakaki G, Fischer R, et al Unexpected deposition patterns of recombinant proteins in post-endospermic reticulum compartments of wheat endosperm. Plant Physiol. 2004; 136: 3457–3466. 15489278 10.1104/pp.104.050153 PMC527145

[26] Rosenberg Y, Sack M, Montefiori D, Forthal D, Mao L, Hernandez-Abanto S, et al Rapid high-level production of functional HIV broadly neutralizing monoclonal antibodies in transient plant expression systems. PloS ONE, 2013; 8(3): e58724 10.1371/journal.pone.0058724 23533588 PMC3606348

[27] Stadlmann J, Pabst M, Kolarich D, Kunert R, Altmann F. Analysis of immunoglobulin glycosylation by LC-ESI-MS of glycopeptides and oligosaccharides. Proteomics 2008; 8: 2858–2871. 10.1002/pmic.200700968 18655055

[28] Kapusi E, Hensel G, Coronado MJ, Broeders S, Marthe C, Otto I, et al The elimination of a selectable marker gene in the doubled haploid progeny of co-transformed barley plants. Plant Mol Biol. 2013; 81: 149–160. 10.1007/s11103-012-9988-9 23180016 PMC3527739

[29] Rechinger KB, Simpson DJ, Svendsen I, Cameron-Mills V. A role for gamma 3 hordein in the transport and targeting of prolamin polypeptides to the vacuole of developing barley endosperm. Plant J. 1993; 4: 841–853. 7506098 10.1046/j.1365-313x.1993.04050841.x

[30] Møgelsvang S, Simpson DJ. Protein folding and transport from the endoplasmic reticulum to the Golgi apparatus in plants. J Plant Physiol. 1998; 153: 1–15.

[31] Wilkinson B, Gilbert HF. Protein disulfide isomerase. Biochim Biophys Acta Protein Proteomics 2004; 1699: 35–44. 10.1016/j.bbapap.2004.02.017 15158710

[32] Arcalis E, Stadlmann J, Marcel S, Drakakaki G, Winter V, Rodriguez J, et al The changing fate of a secretory glycoprotein in developing maize endosperm. Plant Physiol. 2010; 153: 693–702. 10.1104/pp.109.152363 20388665 PMC2879800

[33] Vamvaka E, Twyman RM, Murad AM, Melnik S, Teh AY, Arcalis E, et al Rice endosperm produces an underglycosylated and potent form of the HIV-neutralizing monoclonal antibody 2G12. Plant Biotechnol J. 2015; 10.1111/pbi.12360 PMC11389169 25845722

[34] Hensel G, Himmelbach A, Chen W, Douchkov DK, Kumlehn J. Transgene expression systems in the Triticeae cereals. J Plant Physiol. 2011; 168: 30–44. 10.1016/j.jplph.2010.07.007 20739094

[35] Patel M, Johnson JS, Brettell RIS, Jacobsen J, Xue GP. Transgenic barley expressing a fungal *xylanase* gene in the endosperm of the developing grains. Mol Breed. 2000; 6: 113–123.

[36] Schuenmann PHD, Coia G, Waterhouse PM. Biopharming the SimpliRED^TM^ HIV diagnostic reagent in barley, potato and tobacco. Mol Breed. 2002; 9: 113–121.

[37] Sack M, Rademacher T, Spiegel H, Boes A, Hellwig S, Drossard J, et al From Gene to Harvest: Insights into upstream process development for the GMP production of a monoclonal antibody in transgenic tobacco plants. Plant Biotechnol J. 2015; accepted 10.1111/pbi.12438 26214282

[38] Sainsbury F, Sack M, Stadlmann J, Quendler H, Fischer R, et al Rapid transient production in plants by replicating and non-replicating vectors yields high quality functional anti-HIV antibody. PLoS One. 2010;5(11):e13976 10.1371/journal.pone.0013976 21103044 PMC2980466

[39] Naqvi S, Ramessar K, Farré G, Sabalza M, Miralpeix B, Twyman RM, et al High-value products from transgenic maize. Biotechnol Adv. 2011;29(1):40–53. 10.1016/j.biotechadv.2010.08.009 20816943

[40] Spök A, Twyman RM, Fischer R, Ma JK, Sparrow PA. Evolution of a regulatory framework for pharmaceuticals derived from genetically modified plants. Trends Biotechnol. 2008 26(9):506–17. 10.1016/j.tibtech.2008.05.007 18676047

[41] Levanony H, Rubin R, Altschuler Y, Galili G. Evidence for a novel route of wheat storage proteins to vacuoles. J Cell Biol. 1992; 119: 1117–1128. 1447291 10.1083/jcb.119.5.1117 PMC2289714

[42] Arcalis E, Stadlmann J, Rademacher T, Marcel S, Sack M, Altmann F, et al Plant species and organ influence the structure and subcellular localization of recombinant glycoproteins. Plant Mol Biol. 2013; 83: 105–117. 10.1007/s11103-013-0049-9 23553222

[43] Vuylsteker C, Cuvellier G, Berger S, Faugeron C, Karamanos Y. Evidence of two enzymes performing the de-N-glycosylation of proteins in barley: expression during germination, localization within the grain and set-up during grain formation. J Exp Bot. 2000; 51: 839–845. 10948209

[44] Van Droogenbroeck B, Cao J, Stadlmann J, Altmann F, Colanesi S, Hillmer S, et al Aberrant localization and underglycosylation of highly accumulating single-chain Fv-Fc antibodies in transgenic Arabidopsis seeds. Proc Natl Acad Sci USA 2007; 104: 1430–1435. 17227846 10.1073/pnas.0609997104 PMC1783127

