# Supplementary material for: Transgenic Production of an Anti HIV Antibody in the Barley Endosperm
Source: PLoS One. 2015 Oct 13;10(10):e0140476. doi: 10.1371/journal.pone.0140476 (PMC4604167; doi:10.1371/journal.pone.0140476)
Supplement: S1 Fig — (DOCX) [file pone.0140476.s001.docx]

***AsGLO1*P**

***AsGLO1*P**

**RB**

**LB**

***HPT***

***35S*T**

***NOS*T**

***SP:gfp:KDEL***

**pGH223 (*d35S*P::*HPT*-*AsGLO1*P*::LeB4:gfp:KDEL*)**

***d35S*P**

***AsGLO1*P**

**RB**

**LB**

***HPT***

***35S*T**

**pGH248 (*d35S*P::*HPT*-*AsGLO1*P*::LeB4:LC 2G12:SEKDEL*)**

***d35S*P**

***NOS*T**

***SP:2G12 LC:SEKDEL***

***AsGLO1*P**

**RB**

**LB**

***HPT***

***35S*T**

***SP:2G12 HC:SEKDEL***

**pGH249 (*d35S*P::*HPT*-*AsGLO1*P*::LeB4:HC 2G12:SEKDEL*)**

***d35S*P**

***NOS*T**

**Supplemental Figure 1**: Schematic representation of T-DNA regions of the binary vectors used for transformation. LB - left border; *d35S*P – *CaMV double 35S promoter*; *HPT* – *HYGROMYCIN PHOSPHOTRANSFERASE* selectable marker gene conferring resistance to hygromycin; *35S*T – *CaMV 35S* terminator; *NOS*T – *Agrobacterium NOPALINE SYNTHASE* terminator; *gfp* – *green fluorescent protein* gene; *AsGLO1*P – oat *GLOBULIN1 promoter*; *SP* – legumin B4 signal peptide; *2G12 LC* – 2G12 mAB light chain; *2G12 HC* – 2G12 mAB heavy chain; *SEKDEL* – ER retention signal; RB – right border.
