# Supplementary figures and images for: Transgenic Production of an Anti HIV Antibody in the Barley Endosperm
Source: PLoS One. 2015 Oct 13;10(10):e0140476. doi: 10.1371/journal.pone.0140476 (PMC4604167; doi:10.1371/journal.pone.0140476)

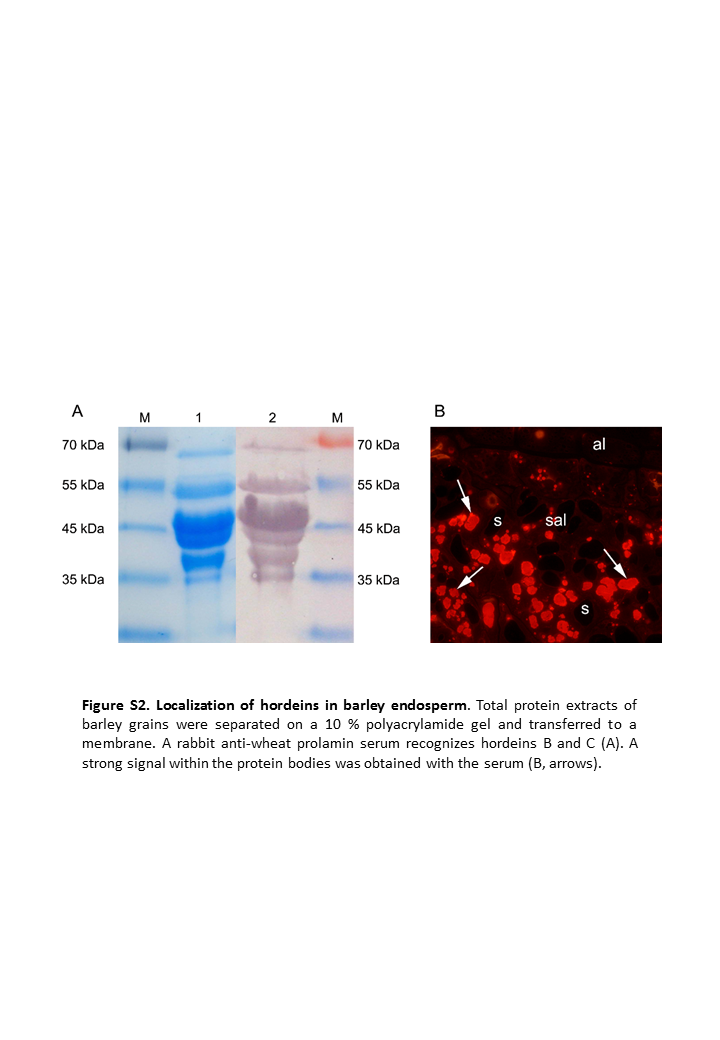

Supplement: S2 Fig — (TIF) [file pone.0140476.s002.tif]
