# Supplementary material for: Transgenic Production of an Anti HIV Antibody in the Barley Endosperm
Source: PLoS One. 2015 Oct 13;10(10):e0140476. doi: 10.1371/journal.pone.0140476 (PMC4604167; doi:10.1371/journal.pone.0140476)
Supplement: S1 Table — (DOCX) [file pone.0140476.s003.docx]

| **Table S1.** Transgene segregation in three T_2_ populations of barley, based on *HPT* expression in germinating immature embryos and GFP quantification in grains of selected plants | | | | | |  |
| --- | --- | --- | --- | --- | --- | --- |
| **T_1_/T_2_ Plants** | **T-DNA copies T_0_ (DNA gel blot)** | **T_2_ IEs analyzed** | **Hygromycin resistant/ susceptible plants** | **Zygosity concluded** | **Amount of sgfp in T_2_/T_3_ [µg/g dry grain]** | **TSP [%]** |
| 1E2-2 | 1 | 40 | inf | - |  |  |
| 1E2-3 |  | 40 | inf | - |  |  |
| 1E2-7 |  | 40 | inf | - |  |  |
| 1E2-10 |  | 40 | inf | - |  |  |
| 1E2-12 |  | 40 | 0:40 | azygous |  |  |
| **1E2-14** |  | **40** | **27:13** | **hemizygous** | **693** | **0.7** |
| **1E2-14-1** |  | **-** | **-** | **-** | **684** | **0.7** |
| **1E2-14-2** |  | **-** | **-** | **-** | **768** | **0.8** |
| 1E2-15 |  | 40 | inf | - |  |  |
| 1E2-16 |  | 40 | inf | - |  |  |
| 1E2-17 |  | 40 | 30:10 | hemizygous |  |  |
| 1E2-18 |  | 40 | 30:10 | hemizygous |  |  |
| 1E12-1 | 1 | 40 | 35:5 | hemizygous |  |  |
| 1E12-3 |  | 40 | 29:11 | hemizygous |  |  |
| 1E12-4 |  | 40 | 35:5 | hemizygous |  |  |
| 1E12-6 |  | 40 | 38:2 | hemizygous |  |  |
| 1E12-9 |  | 40 | 35:5 | hemizygous |  |  |
| **1E12-10** |  | **40** | **40:0** | **homozygous** | **598** | **0.6** |
| 1E12-11 |  | 40 | 37:3 | hemizygous |  |  |
| 1E12-12 |  | 40 | 35:5 | hemizygous |  |  |
| 1E12-15 |  | 40 | 33:7 | hemizygous |  |  |
| 1E12-16 |  | 40 | 30:10 | hemizygous |  |  |
| **1E17-1** | **2** | **40** | **39:1** | **homozygous** | **808** | **0.8** |
| 1E17-2 |  | 39 | 38:1 | homozygous |  |  |
| 1E17-3 |  | 36 | 27:9 | hemizygous |  |  |
| **1E17-4** |  | **40** | **40:0** | **homozygous** | **777** | **0.8** |
| 1E17-5 |  | 40 | 35:5 | hemizygous |  |  |
| 1E17-6 |  | 40 | 36:4 | hemizygous |  |  |
| 1E17-11 |  | 39 | 37:2 | hemizygous |  |  |
| 1E17-13 |  | 40 | 35:5 | hemizygous |  |  |
| 1E17-14 |  | 40 | 37:3 | hemizygous |  |  |
| 1E17-15 |  | 40 | 37:3 | hemizygous |  |  |

inf – boxes with plants were infected and could not be used
