# Supplementary material for: Transgenic Production of an Anti HIV Antibody in the Barley Endosperm
Source: PLoS One. 2015 Oct 13;10(10):e0140476. doi: 10.1371/journal.pone.0140476 (PMC4604167; doi:10.1371/journal.pone.0140476)
Supplement: S2 Table — (DOCX) [file pone.0140476.s004.docx]

**Table S2:** Quantification of ^barley^2G12 antibody in DH lines.

| **Transgenic DH line** | **T-DNA copies**  **(DNA gel blot)** | **% TSP*** |
| --- | --- | --- |
| **BG208/1E05-P4** | 2 | 0.2 |
| **BG208/1E05-P5** | 2 | 0.1 |
| **BG208/1E05-P7** | 2 | 0.3 |
| **BG208/1E05-P14** | 2 | 0.4 |
| **BG208/1E05-P15** | 2 | 0.2 |
| **BG208/1E06-P4** | n.d. | 0.3 |
| **BG208/1E06-P7** | n.d. | 0.4 |
| **BG208/1E06-P13** | 2 | 0.3 |
| **BG208/1E06-P16** | 2 | 0.2 |
| **BG208/1E06-P25** | 2 | 0.4 |
| **BG208/2E07-P1** | 3 | 0.3 |
| **BG208/2E07-P2** | 4 | 0.3 |
| **BG208/2E07-P3** | 4 | 0.3 |
| **BG208/2E07-P5** | 3 | 0.1 |

*% TSP has been roughly estimated by comparison of 3 different concentrations of ^barley^2G12 with 4 standard concentrations (^CHO^2G12)
