# Supplementary material for: Transgenic Production of an Anti HIV Antibody in the Barley Endosperm
Source: PLoS One. 2015 Oct 13;10(10):e0140476. doi: 10.1371/journal.pone.0140476 (PMC4604167; doi:10.1371/journal.pone.0140476)
Supplement: S3 Table — (DOCX) [file pone.0140476.s005.docx]

**Table S3**. Primer sequences used for the identification of T-DNA elements.

| **Primer** | **Sequence 5’ – 3’** | **Amplified region** |
| --- | --- | --- |
| GH-AsGlo1-EcoRV-F1 | gcgatatcTGGAAAGTCATTTTGCCTCCTG | *AsGLO1* promoter – forward primer |
| GH-AsGlo1-PstI-R1 | cgactgcagGAGATTGTAGAAGGTGGATTGGTGC | *AsGLO1* promoter – reverse primer |
| HC-Nco | ccttccatggtgGAGGTGCAGCTGGT | 2G12 heavy chain – forward primer |
| HC-Sal | cccactgtcgactcacagctcgtccttctcgctagccacacgcgtTTTACCCGGAGACAGG | 2G12 heavy chain – reverse primer |
| LC-Nco | ccttccatggtgGATGTTGTGATGACTCAGTC | 2G12 light chain – forward primer |
| LC-Sal | cccactgtcgactcacagctcgtccttctcgctagccacacgcgtACACTCTCCCCTGTTGAA | 2G12 light chain – reverse primer |
| HC-forward | TTCCCCCTGGCACCCTCCTC | 2G12 heavy chain – forward primer |
| HC-reverse | TCCTCCCGCGGCTTTGTCTT | 2G12 heavy chain – reverse primer |
| LC-forward-2 | ACGAACTGTGGCTGCACCATCTG | 2G12 light chain – forward primer |
| LC-reverse-2 | CACTCTCCCCTGTTGAAGCTCTTTG | 2G12 light chain – reverse primer |
| GH-HPT-F1 | TATCGGCACTTTGCATCGGC | *HPT* gene – forward primer |
| GH-HPT-R2 | GATCGGACGATTGCGTCGCA | *HPT* gene – reverse primer |
